# Informal payments by patients, institutional trust and institutional asymmetry

**DOI:** 10.3389/fpsyg.2022.1015208

**Published:** 2022-10-20

**Authors:** Adrian V. Horodnic, Colin C. Williams, Claudia Ioana Ciobanu, Daniela Druguș

**Affiliations:** ^1^Faculty of Medicine, “Grigore T. Popa” University of Medicine and Pharmacy, Iași, Romania; ^2^Management School, University of Sheffield, Sheffield, United Kingdom; ^3^Faculty of Civil Engineering and Building Services, Gheorghe Asachi Technical University of Iași, Iași, Romania

**Keywords:** trust, informal payments, public healthcare service, institutional asymmetry, formal institutions, informal institutions

## Abstract

The aim of this paper is to evaluate the extent of the practice of using informal payments for accessing the services of public clinics or hospitals across Europe and to explain the prevalence of this corrupt practice using the framework of institutional theory. To achieve this, a multi-level mixed-effect logistic regression on 25,744 interviews undertaken in 2020 with patients across 27 European Union countries is conducted. The finding is that the practice of making informal payments remains a prevalent practice, although there are large disparities in the usage of this practice in different European countries. However, informal payments by patients are more likely when there is a lower institutional trust and a higher degree of asymmetry between formal and informal institutions. The resultant proposal is that policy makers need to address the institutional environment to tackle such informal payments. How this can be achieved is outlined.

## Introduction

For many decades the phenomenon of informal payments by patients was thought to be disappearing in the context of economic development and the modernization of health authorities and a minor practice. However, the starting point of this paper is recent data from Kantar (2020), amid the COVID-19 pandemic, when an investigation into informal payments across different sectors in the European Union revealed that the highest share of informal payments (6% of all respondents) was in health services (Kukutschka, 2021), and is connected to the social-economic environment (Balabanova and McKee, 2002; Wamsiedel, 2022a,b). Such informal payments by patients can be seen as “gratitude payments” or “unofficial fees” (Ensor, 2004) that patients offer for receiving preferential access to public health services. It is also referred to “under-the-table payments” (Delcheva et al., 1997), “envelope payments” or “bribes/bribe payments” (Cherecheş et al., 2013). These informal payments by patients can be initiated either by the patients who believe that they will receive more attention and better treatment (Gaal et al., 2006) or by the employees of public healthcare services (Balabanova and McKee, 2002; Jaminson et al., 2006).

Previous studies evaluated the amplitude of the phenomenon, the socio-demographic characteristics of those more inclined to make informal payments as well as the factors driving the informal payments. Starting with its amplitude in the health system, the finding is that this practice is more prevalent in the East-Central Europe region (12%) compared with Nordic nations (1%), with the highest rates occurring in Romania (22%) and Bulgaria, Hungary and Lithuania (19%) (Kukutschka, 2021; Transparency International, 2021). Studies have also sought to explain the disparities across countries or regions (Balabanova and McKee, 2002; Gaal and McKee, 2004; Liaropoulos et al., 2008; Baji et al., 2012; Riklikiene et al., 2014; Williams and Franic, 2016; Stepurko et al., 2017; Williams and Horodnic, 2017, 2018a,b; Horodnic et al., 2018a, 2021. These studies cover drivers belonging to both the formal and informal institutional environments. Starting with the formal institutions’ failures and imperfections, four main categories of drivers of participation to informal practices have been identified by previous studies, namely: resource misallocations and inefficiencies, voids and weaknesses, powerlessness and instability and uncertainty (Williams, 2017). Indeed, determinants from most of these categories have been identified when focusing on the practice informal payments by patients. As such, the voids and weaknesses of the formal institutions include examples of excessive bureaucracy and many laws and regulations (Polese, 2014). Determinants related to resource misallocation and inefficiencies include: lack of transparency (Stepurko et al., 2015; Horodnic et al., 2021), the inefficiency of the health management system (Lewis, 2007; Stepurko et al., 2015; Burnett et al., 2016), the lack of financial resources (Williams and Horodnic, 2018a), a poor health system performance (Tambor et al., 2013; Horodnic et al., 2018b, 2021), or the influence of poor economic performance (i.e., public expenditure on health) and governance performance (Tambor et al., 2013; Stepurko et al., 2015; Incaltarau et al., 2021). Similarly, other determinates of informal payments are related to formal institutions powerlessness exemplified by a low level of penalties imposed on citizens who initiate unofficial payments (Lewis, 2007) and a diminished activity of monitoring the health system (Lewis, 2007).

Meanwhile, studies on the informal institutional environment have revealed the influence of personal factors such as the patients’ beliefs, attitudes, and feelings related to what represents an acceptable behavior or a different perception of the patient-doctor relationship (Horodnic, 2021; Incaltarau et al., 2021). These factors are found to be more relevant at country level (Balabanova and McKee, 2002; Gaal and McKee, 2004; Liaropoulos et al., 2008; Baji et al., 2012; Riklikiene et al., 2014; Williams and Franic, 2016). Similarly, a recent study reveals the association between the prevalence of the informal payments by patients and the lack of alignment between formal and informal institutions (also known in the literature as institutional asymmetry) as underlying the disparities between countries (Horodnic and Williams, 2018). Indeed, recent research emphasizes the relationship between informal payments, corruption, and institutional trust or the trust in public authorities (Horodnic et al., 2021; Incaltarau et al., 2021; Gozgor, 2022).

However, no previous research on this issue has included all the EU countries (wide range of countries, with different levels of development, health system performance etc.) to analyze the link between informal payments by patients and both institutional trust (i.e., trust in public authorities) as well as institutional asymmetry. Therefore, this paper aims to advance understanding by evaluating the influence of institutional determinants on informal payments across all EU countries.

The rest of the paper is structured as follows: the next section briefly synthetizes the findings of the previous research on the determinants found to be relevant in previous literature investigating informal payments by patients to build hypotheses to be tested. Section two then describes the methodology, the materials and data used for testing the hypotheses. The results are reported in the third section. Section four summarizes the findings followed by a discussion on the main policy implications of the results obtained.

## Literature review and hypotheses development

### Institutional trust

Trust has a multitude of facets and has been investigated by scholars from various disciplines. In the field of sociology, previous research focused on explaining what trust is, the types or targets of trust, the functions of trust, the foundations of trust, the mechanism of creating or destroying trust, the origins, determinants and outcomes of trust and social capital (e.g., Fukuyama, 1995; Putnam, 1995; Sztompka, 2003). Researchers in psychology and social psychology have investigated whether trust is an individual disposition or a psychological state to accept vulnerability based on expectations of the behavior of others, whether trust is a personality trait and how trust judgements are made and its dynamics using game theories (e.g., Rousseau et al., 1998; Evans and Revelle, 2008; Freitag and Bauer, 2016; Liu and Chen, 2022). Meanwhile, studies from economics, management and political sciences have focused on institutional trust reflecting the functioning of the overall political legal and economic framework as well as its informal institutions and tries to answer what generates trust in a state/ institution/ organization or what a trustworthy state/institution/organization represents (e.g., Hardin, 2002; Warren, 2004; Welter and Smallbone, 2006). As such, from a social perspective, trust is a vital element that can explain the connection between individuals and government which can play a substantial role in promoting social cohesion during difficult periods (Devine et al., 2021; Gozgor, 2022). From a political perspective, trust in authorities is seen as a necessary condition for obtaining public cooperation and compliance qq(Van Bavel et al., 2020; Devine et al., 2021). Employing the lenses of the institutional theory in informal economy, the level of trust in the authorities represents an important driver explaining peoples’ decision when they choose to make informal payments (Williams and Bezeredi, 2017; Horodnic and Williams, 2018). Other research highlights a negative relationship between social trust and compliance (Goldstein and Wiedemann, 2022).

For the healthcare sector, previous studies discover that informal payments arise when people lose their trust or have a low level of trust in the public system (Pourtaleb et al., 2020; Kukutschka, 2021). Furthermore, previous research highlights that trust is indirectly associated with corruption (Neerup Handlos et al., 2016). Indeed, corruption has been found to have a negative influence on the level of trust in formal institutions (e.g., government) (Kumlin et al., 2018; Todor, 2018; Horodnic and Williams, 2019).

Thus, trust is an important driver that can explain informal payments in the public healthcare sector. Therefore, in order to evaluate the significance of institutional trust, the following hypothesis is proposed:

*H1*: Patients are more inclined to make informal payments when they display a lower level of trust in authorities.

### Institutional asymmetry

Previous research underlines that patients’ behaviors seem to be shaped by the institutional environment in which they are embedded (Scott, 2008). Indeed, in all societies the institutional environment is shaped by both formal and informal rules. Generally, an institution can be seen as a set of rules respected by the citizens of a country (Denzau and North, 1994; Mathias et al., 2014). Formal institutions are the written codified rules and informal institutions are the “socially shared rules, usually unwritten, that are created, communicated and enforced outside of officially sanctioned channels” (Helmke and Levitsky, 2004, p. 727). Seen through the institutional theory lens, early research has viewed formal institutional failures as explaining the prevalence of informal payments in the healthcare system (Lewis, 2002; Ensor, 2004).

Later, the institutional framework for the healthcare system has depicted the complex issue of institutional asymmetry caused by a lack of alignment of the codified rules of formal institutions to the norms, values and beliefs or the unwritten rules of informal institutions (Baumol and Blinder, 2008; Williams and Horodnic, 2017, 2018a,b; Kukutschka, 2021). As such, rooted in a variant of the institutional theory developed by North (1990), informal payments made by patients are seen to have a close relationship to the asymmetry between the formal and informal rules. Informal payments appear to constitute an attempt to escape formality and follow common informal unwritten rules that guide patients’ behavioral patterns. As such, to explain the prevalence of informal payments, understanding this institutional asymmetry process and its determinants is required.

Previous research reveals several systemic factors that explain this institutional asymmetry. The structural conditions related to failures of the formal institutional environment leading to a higher widespread of informal payments include: economic determinants (i.e., low allocation level for public health expenditure) or poor government performance (Cohen, 2012; Tambor et al., 2013; Stepurko et al., 2015). Other studies have identified the influence of the formal institutional imperfections (voids) and formal institutions inefficiencies (Horodnic and Williams, 2018; Incaltarau et al., Kukutschka, 2021) on the level of institutional asymmetry and therefore, the extent of the informal payments. They also reveal the influence of institutional imperfections such as the lower levels of expenditure on healthcare (Balabanova and McKee, 2002; Burnett et al., 2016) or an inadequate budget allocation for healthcare services (Gaal and McKee, 2004; Kutzin et al., 2010; Baji et al., 2012; Tomini et al., 2012a) as well as inefficiencies such as the low level of government performance, the low quality of healthcare system (Lewis, 2002; Gaal and McKee, 2004; Rechel et al., 2011; Tambor et al., 2013; Tomini and Groot, 2013; Horodnic et al., 2021) or corruption (Williams and Horodnic, 2017). Indeed, synthesizing the previous findings in literature, Williams (2017) shows that there is a link between various forms of corruption (such as bribes, state capture or the use of personal connections) and the level of institutional asymmetry.

Nevertheless, these drivers will have a different signification for different countries, due to different levels of development. For instance, in post-communist transition economies, where the level of development of the public services system is poor and corruption practices are prevalent, informal payments occur more often and the institutional asymmetry approach is more relevant (Williams, 2017; Williams and Horodnic, 2018b). For example, research conducted in 2010 by Stepurko et al. (2015) in Central and Eastern regions of Europe indicates Romania (35%) and Lithuania (25%) as countries where informal payments are more prevalent. Other research, on 11 countries of the same region based on a Eurobarometer survey undertaken in 2021, show Romania (22%) followed by Bulgaria (19%), Hungary (19%), Lithuania (19%) and Croatia (15%) as countries where informal payments occur more often (Horodnic et al., 2021).

However, not only the shortcomings of the formal institutions influence the distribution of the informal payments by patients. Indeed, previous research shows that informal institutions play an important role in determining the behavior of the individuals. For example, ethical aspects and social custom of showing appreciation by paying informally also play a role in shaping individuals’ behavior (Stepurko et al., 2015; Williams and Horodnic, 2018a,b). Indeed, despite the shortcomings of the formal institutions, the informal payments do not occur when informal institutions are aligned to the formal institutions (Williams and Horodnic, 2018b). As such, if the informal institutions are “complementary” and support the rules set by the formal institutions, the practice of paying informally for public medical services does not occur despite the weaknesses of the formal institutional environment. However, when the informal institutions are “substitutive” to the formal institutions and prescribe discordant rules, the practice of informal payments occur. As such, these payments only occur when there is a misalignment between the informal and formal institutions which results in perceiving this type of payment as legitimate and acceptable (Williams and Horodnic, 2018b). Thus, the following hypotheses is proposed:

*H2*: Patients are more inclined to make to make informal payments when they display a greater degree of institutional asymmetry.

## Materials and methods

To evaluate the relationship between the prevalence of informal payments, institutional trust and institutional asymmetry, we use data collected from 27 Member states of the European Union (EU-27) for the 2021 Global Corruption Barometer (GCB). The survey was applied to a number of 40,663 respondents, of which 25,744 respondents had used the public health services in the past 12 months before the survey. Corruption practices were the main subject of this survey. The respondents are adults over 18 years old, and the sample is representative at regional level as it contains a minimum of 300 respondents (for NUTS 1 level), according to Eurostat’s Nomenclature. The sample design ensures that the variables related to gender, age, social status, and educational level reflect the whole population parameters (for details see Kantar, 2020).

Accounting for the hierarchical structure of the data (individuals clustered in countries) and for the country effect, a multilevel logistic regression analysis has been employed. The dependent variable indicates if the respondent made informal payments or not before the survey (in the past 12 months), and it is a dichotomous one.

The independent variables used for testing the proposed hypotheses are:

- Institutional Trust Index – a measure of the institutional trust based on individuals’ self-assessed level of trust in local and national authorities. The score has been obtained as an average of the self-assed level of trust in local authorities and the self-assed trust in national authorities (for testing Hypothesis 1);
- Institutional Asymmetry – a variable measuring whether or not the legal rules of formal institutions are in line with the norms and values of the informal institutions by investigating the acceptability of the citizens towards corruption acts from the government authorities in the event of delivering good outcomes (for testing Hypothesis 2).

The indexes were normalized using a 0–1 scale, where 1 is associated with positive outcome (i.e., a high trust in public institutions, a high level of alignment between the rules of formal and informal institutions) and 0 is associated with undesirable outcome. A lower value of the indices is therefore associated with less trust and a higher level of institutional asymmetry.

The independent variables used as control variables are chosen in accordance to the specifications of previous research on trust, the informal economy and informal payments for health services (Balabanova and McKee, 2002; Szende and Culyer, 2006; Tomini and Maarse, 2011; Tomini et al., 2012a,b; Baji et al., 2013; Kaitelidou et al., 2013; Tomini and Groot, 2013; Riklikiene et al., 2014; Arsenijevic et al., 2015; Danyliv et al., 2015; Stepurko et al., 2015; Williams et al., 2016; Ai et al., 2022; Horodnic et al., 2022a) and include: age, gender, educational level, income, employment status, residence area (rural or village) (details in Table 1).

**Table 1:** Multilevel logistic regression of the patient’s likelihood to make informal payments in EU-27: the role of institutional trust and institutional asymmetry.

	Null model				Model 1					Model 2			
Fixed part	Coef.		SE		Coef.		SE	OR		Coef.		SE	OR
Control variables													
Female					0.079		0.062	1.083		0.062		0.059	1.064
Age					−0.011	***	0.002	0.989		−0.011	***	0.003	0.990
Tertiary education					0.063		0.063	1.065		0.095		0.060	1.099
Employments status (R: Working full-time)													
Working part-time					0.070		0.078	1.072		0.078		0.085	1.081
Not working (seeking)					−0.105		0.094	0.901		−0.129		0.102	0.879
Retired					−0.105		0.094	0.900		−0.105		0.101	0.901
Not working (not seeking)					−0.203		0.177	0.816		−0.188		0.176	0.829
Student					−0.031		0.108	0.969		−0.024		0.103	0.976
Homemaker					0.039		0.173	1.040		0.018		0.180	1.018
Household income (R: Enough to buy what wanted)													
Enough to buy what needed					−0.005		0.056	0.995		0.018		0.048	1.019
Facing difficulties					0.129	***	0.049	1.137		0.146	***	0.051	1.157
Area (R: Rural area or village)													
Small, middle-sized town					0.031		0.080	1.031		0.046		0.083	1.047
Large town					0.081		0.074	1.085		0.092		0.081	1.096
Institutional trust and asymmetry													
Institutional trust index^1^					−1.252	***	0.100	0.286		−1.279	***	0.103	0.278
Institutional asymmetry^2^										−0.366	***	0.064	0.693
Constant	−1.286	***	0.029		−0.521	***	0.181			−0.326	**	0.166	
Random part													
Country-level variance	5.930							4.555		4.172			
(Standard error)	1.290							1.078		0.969			
Variance: country level (ICC) (%)	64							58		56			
Observations	25,644							24,958		24,332			
Groups (countries)	27							27		27			
*F*								21.92		31.58			
Prob. > F								0.000		0.000			

Significant at *p < 0.1, **p < 0.05, ***p < 0.01; For categorical variables – coefficients are compared to the reference category in brackets (R).

1 Institutional trust index: 0 (low institutional trust) to 1 (high institutional trust).

2 Institutional asymmetry: 0 (high institutional asymmetry) to 1 (low institutional asymmetry).

Source: author’s own work.

To apply the analysis, two stages were necessary. In the first stage the necessity of using a multilevel regression approach was verified. As such, an estimation of the baseline random intercept model without independent variables was conducted. The null hypotheses assumes that there is no significant variance by country regarding who makes informal payments in order to access public health services. The likelihood-ratio test rejected the lack of variation and indicate the need of using the multilevel models. Indeed, the results of the null model presented in Table 1 point out that 64% variance in the patient’s predilection to make informal payments was registered at EU country level (Wald = 21.13, df = 1, *p* < 0.001) showing that there are significant differences between countries when analyzing the patient’s propensity to make informal payments for accessing healthcare services provided a public clinic or hospital. Thus, the multilevel logistic regression is required.

An additive approach was used in the second stage of the methodological framework. For constructing the multilevel logistic regression final model, the socio-demographic variables of the individuals, the institutional trust index and institutional asymmetry variable were added in turn, in order to evaluate their effect on the patient’s likelihood to make informal payments. In addition, a graphic representation has been provided of the predicted probabilities of patients to make such payments by the level of institutional trust and institutional asymmetry for enabling understanding of the results. The results of the analysis are presented below.

## Results

### Descriptive analysis

Table 2 presents an overview of the cross-country variations in informal payments made by individuals for accessing public health care services across the EU Member States. Some 25,744 individuals stated that they used public health services in the past 12 months prior the survey from a total of 40,663 participants. The practice of making informal payments is found to be more prevalent in Romania (22% of users of health care services) which is followed by Bulgaria, Hungary and Lithuania (19%). It is least prevalent as a practice in Denmark, Finland, Ireland, Luxembourg, Netherlands, Spain, and Sweden. However, analyzing how often patients resort to informal payments (patients declaring that they make such payments) there are also high variations (Table 2). For example, in Netherlands where this practice is not so prevalent (1%) this behavior occurs on a more regular basis (36% of those paying informally, which is a greater value than the average of 15% for all 27 EU countries or than the 26% in Romania where this practice is more prevalent). This is similarly the case for other countries having a low prevalence of such payments, like Cyprus and Denmark. In these countries, for those making such payments, this is a regular practice when accessing a public healthcare service (34 and 32%, respectively, of those making such payments). Informal payments are rather an exception in Sweden and Malta, being made only once or twice (100% of those making such payments). Similarly, a high number of those making informal payments did so rarely in Belgium (95%), Lithuania (94%) or Poland (93%).

**Table 2:** Informal payments by patients, institutional trust, and institutional asymmetry (*n* = 25,744).

Country	Informal payments by patients	Informal payments frequency		Institutional trust index^1^	Institutional asymmetry^2^
		Rarelly^3^	Often		
	(%)	(%)	(%)	(mean)	(mean)
Austria	6	86	14	0.70	0.87
Belgium	7	95	5	0.58	0.84
Bulgaria	19	85	15	0.34	0.79
Croatia	15	76	24	0.32	0.66
Cyprus	3	66	34	0.33	0.80
Czechia	10	95	5	0.50	0.84
Denmark	1	68	32	0.59	0.94
Estonia	2	89	11	0.57	0.75
Finland	1	77	23	0.62	0.88
France	2	87	13	0.60	0.87
Germany	2	90	10	0.69	0.90
Greece	10	71	29	0.41	0.77
Hungary	19	77	23	0.55	0.72
Ireland	1	85	15	0.59	0.89
Italy	3	81	19	0.51	0.85
Latvia	10	81	19	0.50	0.69
Lithuania	19	94	6	0.47	0.55
Luxembourg	1	89	11	0.67	0.88
Malta	4	100	0	0.58	0.84
Netherlands	1	64	36	0.61	0.86
Poland	10	93	7	0.37	0.72
Portugal	2	98	2	0.55	0.88
Romania	22	74	26	0.36	0.40
Slovakia	10	94	6	0.50	0.86
Slovenia	5	96	4	0.49	0.81
Spain	1	99	1	0.39	0.88
Sweden	1	100	0	0.62	0.94
EU-27 - Total	6	85	15	0.52	0.81

1 Institutional trust index: 0 (low institutional trust) to 1 (high institutional trust).

2 Institutional asymmetry: 0 (high institutional asymmetry) to 1 (low institutional asymmetry).

3 Once, twice, or few times.

Source: author’s own work.

Table 2 also starts to evaluate the relationship between the informal payments’ prevalence, institutional trust, and institutional asymmetry. The finding is that in those countries where it is less likely to make such payments there is a higher Institutional Trust Index (i.e., a high trust in government and other state authority). In the countries where informal payments are not so prevalent (1%) the value registered is higher than the average of all 27 EU Countries of 0.52 (0.59 for Denmark; 0.62 for Finland; 0.59 for Ireland; 0.67 Luxemburg; 0.61 Netherlands; and 0.62 Sweden). For other countries with a high prevalence of this practice such as Romania, Bulgaria or Lithuania, there is a low trust in public authorities as the value of the Institutional Trust Index is lower than the average in EU 27 Member States (0.36 for Romania; 0.34 for Bulgaria and 0.47 for Lithuania). Only for Hungary is the value of the Institutional Trust Index very close to the average registered across all the 27 EU countries; 0.55 compared to 0.52). In sum, those countries with a lower prevalence of informal payments by patients (Denmark, Finland, Ireland, Luxembourg, Netherlands, Spain, and Sweden) register a higher Institutional Trust Index compared to those with a large share of informal payments (i.e., Romania, Bulgaria or Lithuania).

When analyzing institutional asymmetry, the results show that those who live in countries with a small share of informal payments, have a lower degree of asymmetry between formal and informal asymmetry by registering higher values of the Institutional Asymmetry (Denmark, 0.94; Finland, 0.88; Ireland, 0.89; Luxembourg, 0.88; Netherlands, 0.86; Spain, 0.88; or Sweden, 0.94). In countries where informal payments are more prevalent such as Romania, Bulgaria, Hungary or Lithuania the registered values for the Institutional Asymmetry are lower than the average value for all 27 EU Member States, displaying a high asymmetry between formal and informal institutions. Therefore, those who live in a country where informal payments are less prevalent (e.g., Denmark, Finland, Ireland, Luxembourg, Netherlands, Spain, and Sweden) have a lower degree of asymmetry between institutions (formal and informal) compared with those who live in countries where this practice is highly prevalent (e.g., Romania, Bulgaria, Hungary or Lithuania).

As such, the tentative descriptive finding is that the institutional trust and the asymmetry between institutions (formal and informal) are directly related to the propensity of offering informal payments for public healthcare services.

### Multivariate analysis

A multilevel logistic regression analysis was conducted to evaluate whether the tentative findings continue when other variables are added. The results are presented in Table 1. To test the reliability of the findings we here use an additive fashion. The analysis starts with the dependent variable alone (null model) and then gradually the socio-demographic variables of the respondents, followed by Institutional Trust Index and Institutional Asymmetry are added in turn. The outcome of the null model displays that 64% of the variance for informal payments by patients is registered at country level. Analyzing the results of Model 1 which tests the influence of socio-demographic variables shows that older patients are less likely to pay informally the medical staff for health care services. Meanwhile, people who are facing financial difficulties are more likely to make informal payments for accessing public health services. For the other characteristics of the respondents (gender, education, employment status or type of living area) no significant differences were identified. Turning to the institutions in Table 1, and analyzing trust in public authorities, the finding is that patients with a lower Institutional Trust Index (i.e., low trust in institutions) are more likely to pay informally for public health care services (validating Hypothesis 1).

Analyzing Model 2, the finding is that those with a higher value of Institutional Asymmetry (low institutional asymmetry) are less likely to pay informally for public health care services (validating Hypothesis 2).

The results of the sensitivity analysis obtained by applying various alternative regression methods are presented in Table 3. They were conducted to evaluate the robustness of all the findings in Table 1. The results presented in the first column of Table 3 represent a synthesis of the multilevel logistic regression (on weighted data) which was detailed in Table 1. However, a similar outcome is obtained if an alternative statistical method is used, namely the multilevel logistic regression with no weighted data or with imputed data for missing values. The same assessment is valid for the similar case of applying a logistic regression clustered by country or when the potential sample selection bias is considered (i.e., not all respondents in the sample used a public healthcare service). Using these alternative methods, the results (significance, direction of the association) for the Institutional Trust Index and Institutional Asymmetry on the likelihood to pay informally remain unchanged.

**Table 3:** Sensitivity tests.

	Multilevel logistic regression			Logistic regression		Probit regression with sample selection	
	Weighted data: Yes	Weighted data: No	Missing data: Imputed	Weighted data: No	Missing data: Imputed	Weighted data: Yes	Weighted data: No
Control variables	Yes	Yes	Yes	Yes	Yes	Yes	Yes
Institutional trust and asymmetry							
Institutional trust index^1^	−1.279*** (0.103)	−1.279*** (0.115)	−1.267*** (0.112)	−1.827*** (0.233)	−1.813*** (0.227)	−0.815*** (0.072)	−0.811*** (0.047)
Institutional asymmetry^2^	−0.366*** (0.064)	−0.366*** (0.061)	−0.386*** (0.061)	−0.800*** (0.156)	−0.806*** (0.159)	−0.312*** (0.038)	−0.350*** (0.027)
Clustered by country				Yes	Yes		
Imputations (multivariate)			Yes		Yes		
Selection equation^3^						Yes	Yes
Observations	24,332	24,327	25,774	24,327	25,774	39,016	39,016
Censored							14,689
Uncensored							24,327
Prob. > F / chi2	0.000	0.000	0.000	0.000	0.000	0.000	0.000

Significant at *p < 0.1, **p < 0.05, ***p < 0.01; sandard errors in brackets.

1 Institutional trust index: 0 (low institutional trust) to 1 (high institutional trust).

2 Institutional asymmetry: 0 (high institutional asymmetry) to 1 (low institutional asymmetry).

3 Socio-demographic variables included in the selection equation: age, education, and region.

Source: author’s own work.

**Figure 1:**
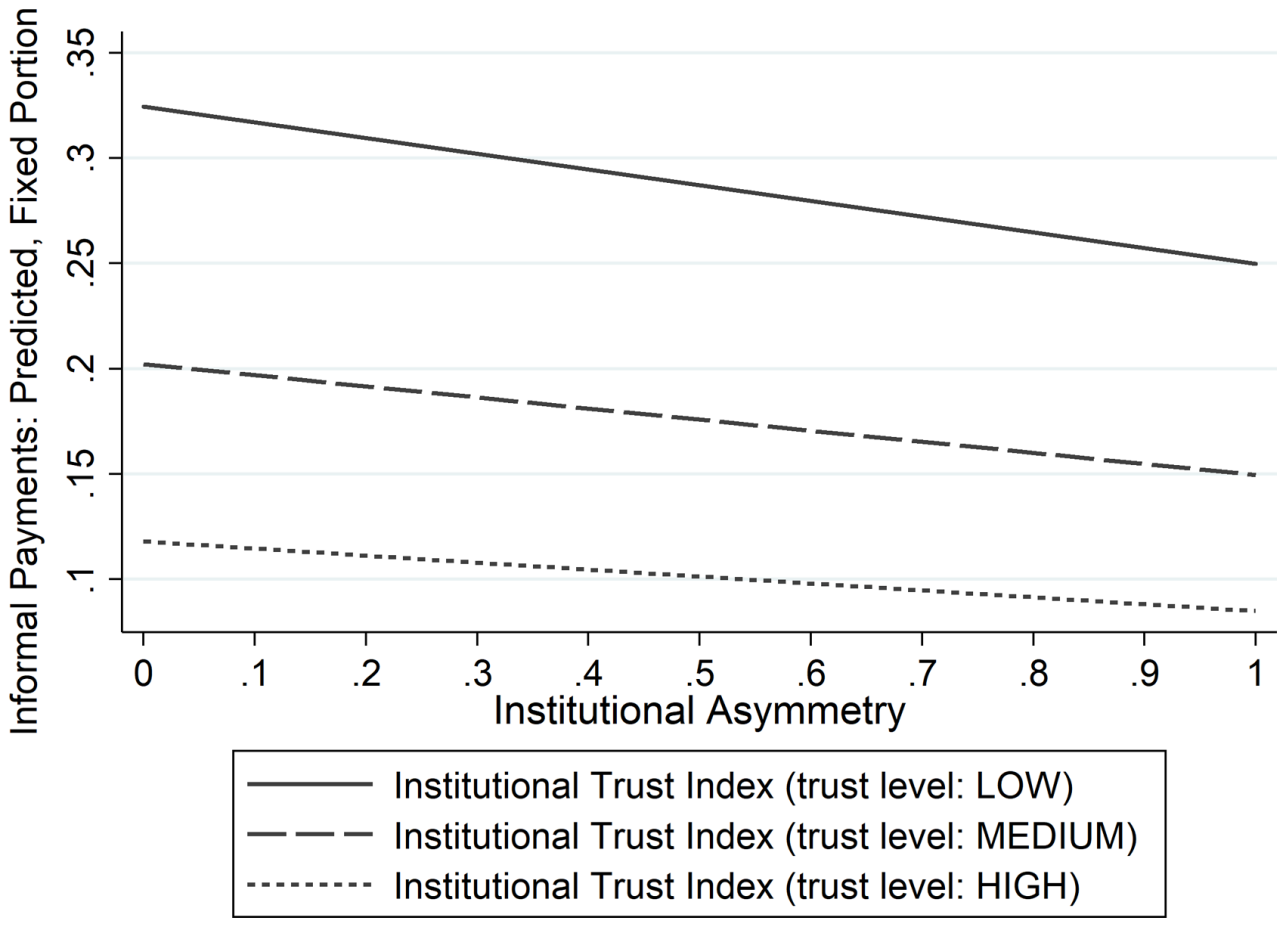
Informal payments by patients: predicted probability, by institutional trust and institutional asymmetry. Calculated after multilevel logistic regression, for a representative patient in the sample: 49 years old female working full time and living in a small or middle-sized town, with primary or secondary education and sufficient money to buy what needed; institutional asymmetry: 0 (high institutional asymmetry) to 1 (low institutional asymmetry). Source: author’s own work.

To understand the findings more easily, a graphic description of the predicted probability of paying informally for public health services for a “representative” patient in Europe according to the Institutional Trust Index and Institutional Asymmetry is presented in Figure 1. The results show that the informal payments share is larger when there is low trust in institutions and a high institutional asymmetry.

## Discussion and conclusions

Using recently collected data, this paper focuses on institutional trust and institutional asymmetry as determinants of informal payments in the health sector. The proposed aim of this paper has been to advance understanding of institutional theory by evaluating if its framework is appropriate for explaining variations across the EU-level in informal payments. While the institutional asymmetry explanation has been previously evaluated in other contexts (e.g., construction services; Horodnic et al., 2022b) and regions (e.g., Central and Eastern Europe or South-eastern Europe; Williams and Horodnic, 2018a,b) and the role of trust in shaping consumer and patient behavior has been extensively evaluated in literature (Kukutschka, 2021; Pop et al., 2022), the institutional trust explanation (trust in institutions) is rarely evaluated as a determinant of informal payments by patients. As such, the influence of institutional trust and institutional asymmetry as determinants of such payments is tested for the first time across all 27 EU countries. The findings show that 6% of the patients in the EU-27 made informal payments in the year prior to survey, with 85% of them rarely making this type of payment. This is in line with the outcome of previous research which finds that committing acts of corruption such as making informal payments are still present despite being rather ignored in this period (Burki, 2019; Horodnic et al., 2021; Gozgor, 2022).

As such, governments should develop a set of measures aimed to prevent and reduce the use of informal payments. To develop such measures, it is vital to identify the category of determinants that generate the occurrence of this characteristics and the socio-economic characteristics of those more likely to engage in such behavior. The results show that younger people and those with financial difficulties should be targeted in public national campaigns as they are found more likely to pay informally for health services. These characteristics of the patient prone to pay informally for accessing the health system across EU member states are in line with previous results, from different regions, that identify young people (Balabanova and McKee, 2002; Tomini and Maarse, 2011; Tomini et al., 2012a; Arsenijevic et al., 2015; Danyliv et al., 2015) or people with low income (Szende and Culyer, 2006; Tomini and Groot, 2013) as being more prone to pay informally.

This paper also provides a more nuanced explanation about the influence of the asymmetry between institutions (formal and informal rules) and institutional trust. As previous studies suggest, people are tempted to behave by following the unwritten laws that make the informal payments acceptable when there is a high degree of institutional asymmetry (formal and informal environment) in their country (Williams and Horodnic, 2017, 2018b; Horodnic et al., 2021). Indeed, this paper reinforces this finding and shows that the institutional asymmetry has a powerful positive influence.

Building trust in formal institutions is also necessary to tackle informal payments. This institutional determinant is even more significant currently when the citizens level of trust in institutions is predisposed to the socio-economic climate (Goldfinch et al., 2021). When the level of trust in institutions (public authorities) is higher, then informal payments are less likely to have a high prevalence. Therefore, measures taken by the public authorities should focus on building peoples’ trust to reduce the gap between institutions (between informal and formal institutions). In each country, the policy measures imposed by the public authorities should analyze both formal and informal environments and also consider the influence of trust in formal institutions as a significant element that leads to the occurrence of informal payments. As such, authorities could organize awareness campaigns or public events that are aimed at changing patient’s behavioral intentions (and thus reducing institutional asymmetry), they can use normative appeals, or even make changes in the formal institutions to tackle the informal payments by patients.

Nevertheless, limitations exist to this study. Due to data availability issues, this analysis could not control for the perceived level of sanctions nor for the risk of being detected when using informal payments to access healthcare services. Future research therefore could focus on addressing these issues. Another limitation of the paper is that the dataset used does not allow to identify the determinants of institutional asymmetry. Future research should employ qualitative methods in order to understand how institutional asymmetry has been generated and how the gap between the formal and informal institutions can be narrowed.

To conclude, this paper has advanced understanding of informal payments in the healthcare system across Europe using the institutional theoretical framework. Recent data has been analyzed and the findings show that low trust in public authorities and a high degree of institutional asymmetry are directly related to the propensity of offering informal payments for public healthcare services. However, future studies could analyze more widely the interdependencies between these factors that influence the informal payments by patients identified in this study. Whether these results are valid for other countries and regions beyond the EU27 needs to be evaluated.

## Data availability statement

Publicly available datasets were analyzed in this study. This data can be found at: https://www.transparency.org/en/gcb/eu/european-union-2021.

## Author contributions

All authors listed have made a substantial, direct, and intellectual contribution to the work and approved it for publication.

## Funding

This work was supported by a grant of the Ministry of Research, Innovation and Digitization, CNCS/CCCDI—UEFISCDI, project number PN-III-P1-1.1-TE-2019-0163, within PNCDI III.

## Conflict of interest

The authors declare that the research was conducted in the absence of any commercial or financial relationships that could be construed as a potential conflict of interest.

## Publisher’s note

All claims expressed in this article are solely those of the authors and do not necessarily represent those of their affiliated organizations, or those of the publisher, the editors and the reviewers. Any product that may be evaluated in this article, or claim that may be made by its manufacturer, is not guaranteed or endorsed by the publisher.
